# Electrosurgical mucosotomy for intramural esophageal dissection

**DOI:** 10.1016/j.vgie.2024.08.012

**Published:** 2024-08-29

**Authors:** Scott MacKay, John Yee, Ananya Nair, Roberto Trasolini

**Affiliations:** 1 Division of General Internal Medicine, Department of Medicine, University of Alberta, Edmonton, Alberta, Canada; 2 Division of Thoracic Surgery, Department of Surgery, University of British Columbia, Vancouver, British Columbia, Canada; 3 Temerty Faculty of Medicine, University of Toronto, Toronto, Ontario, Canada; 4 Division of Gastroenterology, Department of Medicine, University of British Columbia, Vancouver, British Columbia, Canada

## Background

Intramural esophageal dissection (IED) occurs when the mucosal and muscular layers of the esophageal wall are separated to form a false lumen.^1^^,^^2^ IEDs are most commonly the result of hematoma and/or abscess formation in the esophageal wall, which can occur spontaneously or after esophageal mucosa penetration.^3^^,^^4^ Patients with IED present with esophageal dysphagia and chest pain, with hematemesis and septic presentations also described in the literature.^4^^,^^5^ Management of IEDs is generally conservative, although endoscopic stenting and incision also have been described previously.^2^^,^^4^^,^^6^, ^7^, ^8^, ^9^ Surgical intervention is reserved for IEDs complicated by esophageal perforation.^2^

## Case

**Figure 1:**
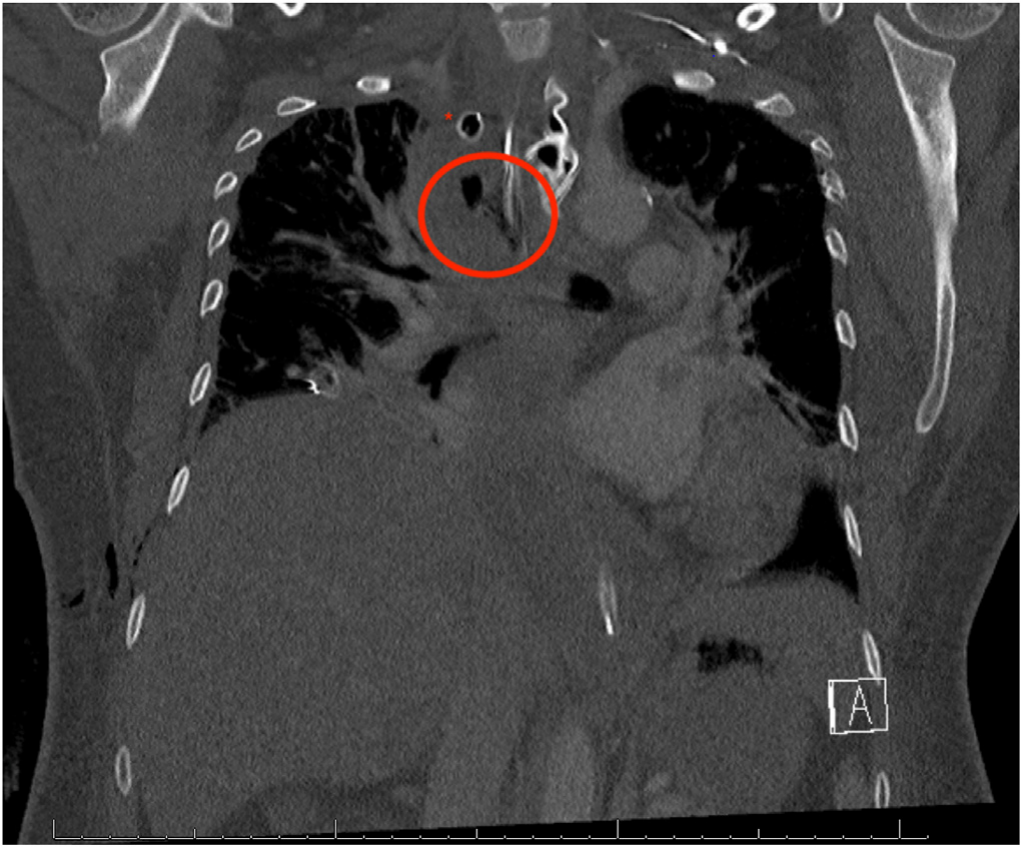
Coronal plane image from a CT scan before intervention by the thoracic surgery department demonstrating the esophageal intramucosal dissection (within the *red circle*) and anterior chest tube (indicated by the *red asterisk*).

A 47-year-old woman presented to the hospital 12 days after reportedly swallowing a fishbone. She reported retrosternal pain and odynophagia. Initial CT imaging demonstrated submucosal edema and thickening of the esophageal wall from the C6 vertebrae to the upper mediastinum. She became increasingly diaphoretic and tachypneic despite the administration of dexamethasone and broad-spectrum antibiotics. A follow-up CT 4 days later demonstrated diffuse esophagitis with associated mediastinitis and new bilateral pleural effusions (Fig. 1). She was transported to a quaternary hospital for urgent intervention for a suspected esophageal perforation.

**Figure 2:**
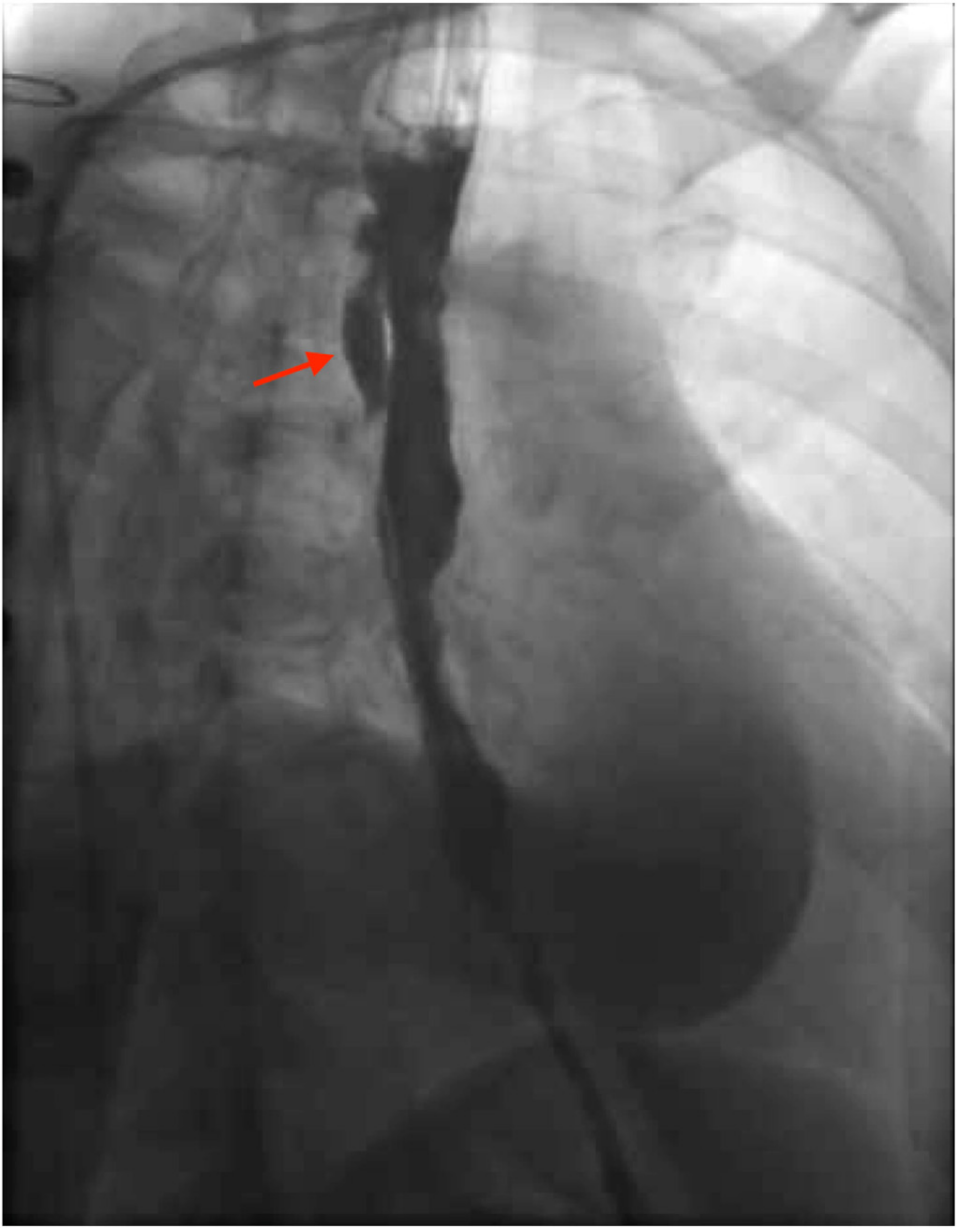
Preprocedure fluoroscopic esophagram demonstrating a false esophageal lumen, indicated by the *red arrow*.

The thoracic surgery department completed esophagoscopy, left-sided neck exploration, and right-side thoracoscopy for drainage of prevertebral and mediastinal collections. Two esophageal mucosal perforations were seen intraoperatively, which were theorized to have been caused by ingestion of the fishbone, although neither were transmural. She was intubated postoperatively and started on postpyloric enteral feeds. Repeat CT on postoperative day 18 showed a new esophageal dissection with a false lumen confirmed on subsequent fluoroscopic esophagram (Fig. 2). She was symptomatic with esophageal dysphagia and a globus sensation once oral intake was resumed. Gastroenterology was consulted regarding this dysphagia with a plan to complete EGD.

## Procedure

**Figure 3:**
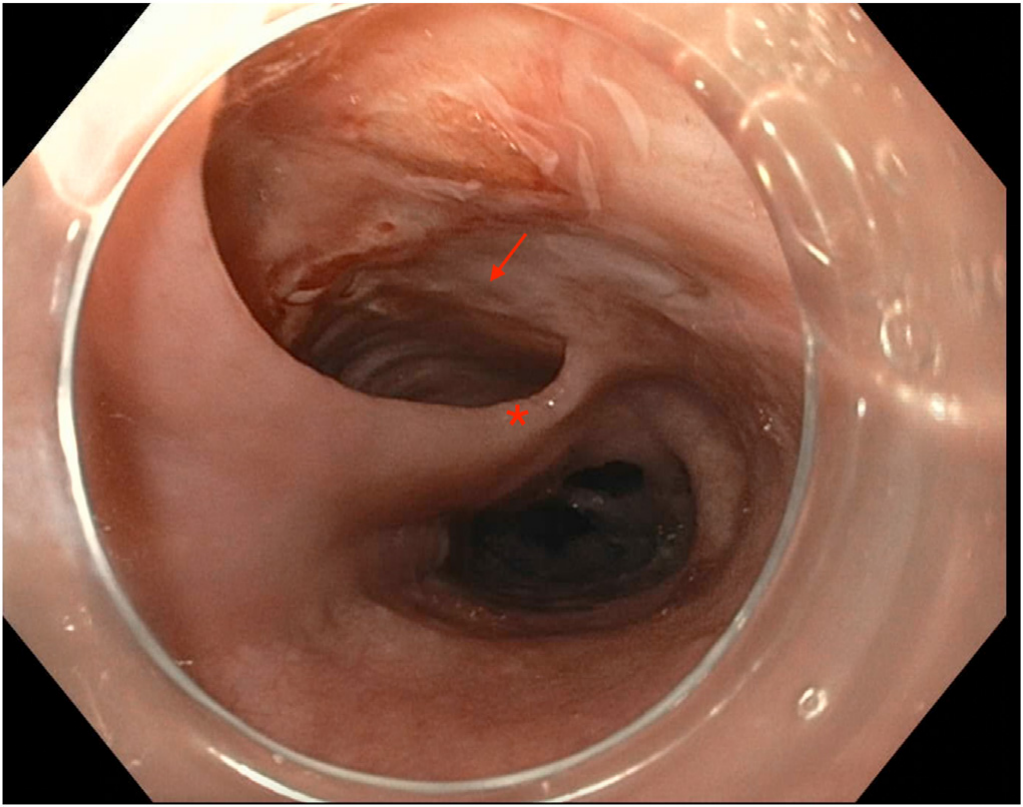
Initial endoscopic view demonstrating a large false esophageal lumen (indicated by the *red arrow*) and mucosal septum dividing true and false esophageal lumens (indicated by the *red asterisk*).

**Figure 4:**
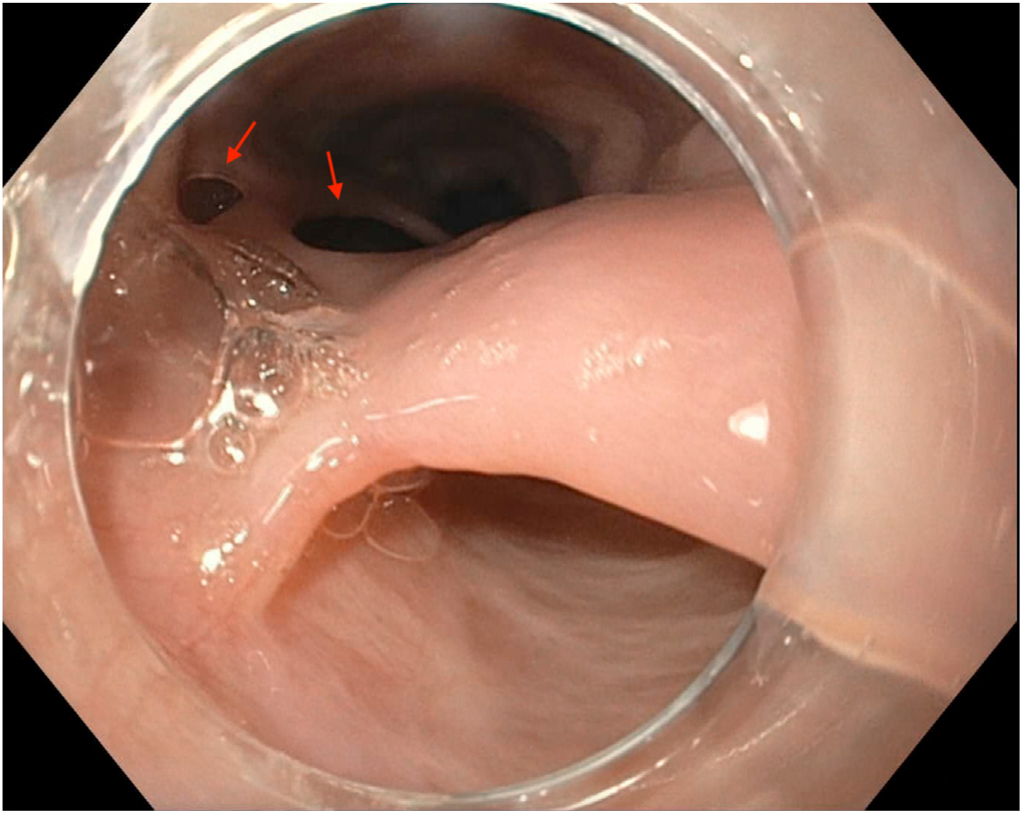
Multiple fenestrations within the mucosal septum (indicated by the *red arrows*).

**Figure 5:**
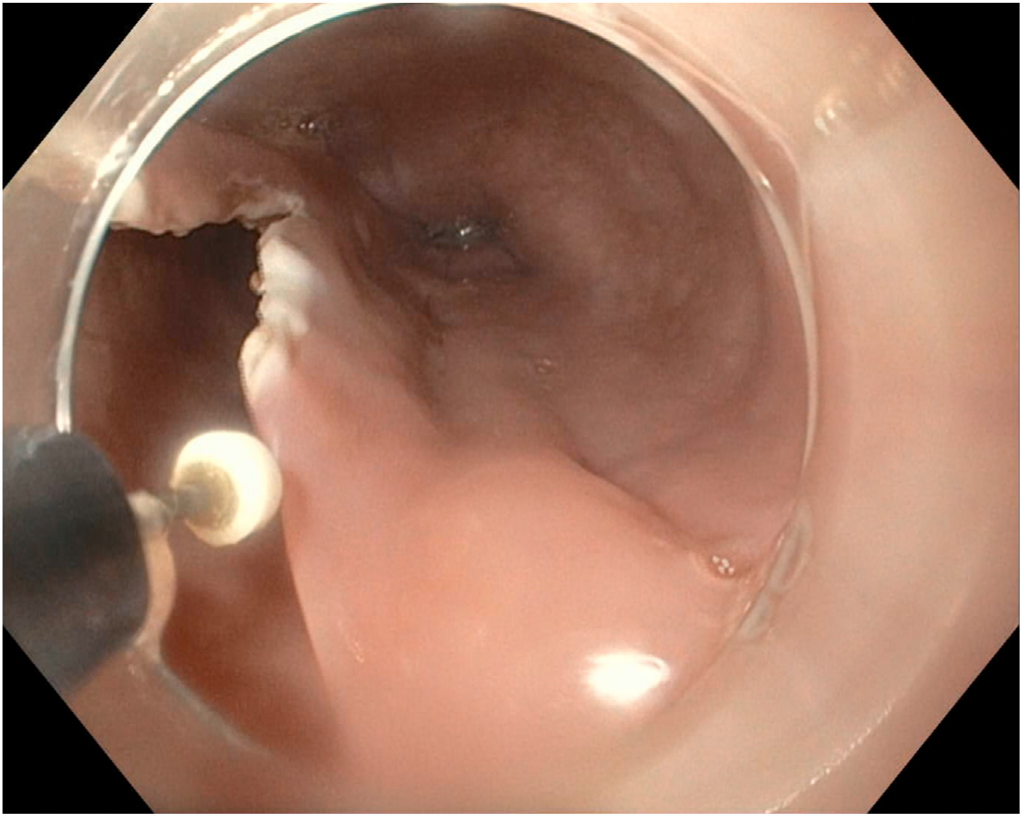
Mucosotomy of the septum was completed endoscopically with an electrosurgical knife. This figure demonstrates initial progress on the proximal aspect of the septum.

**Figure 6:**
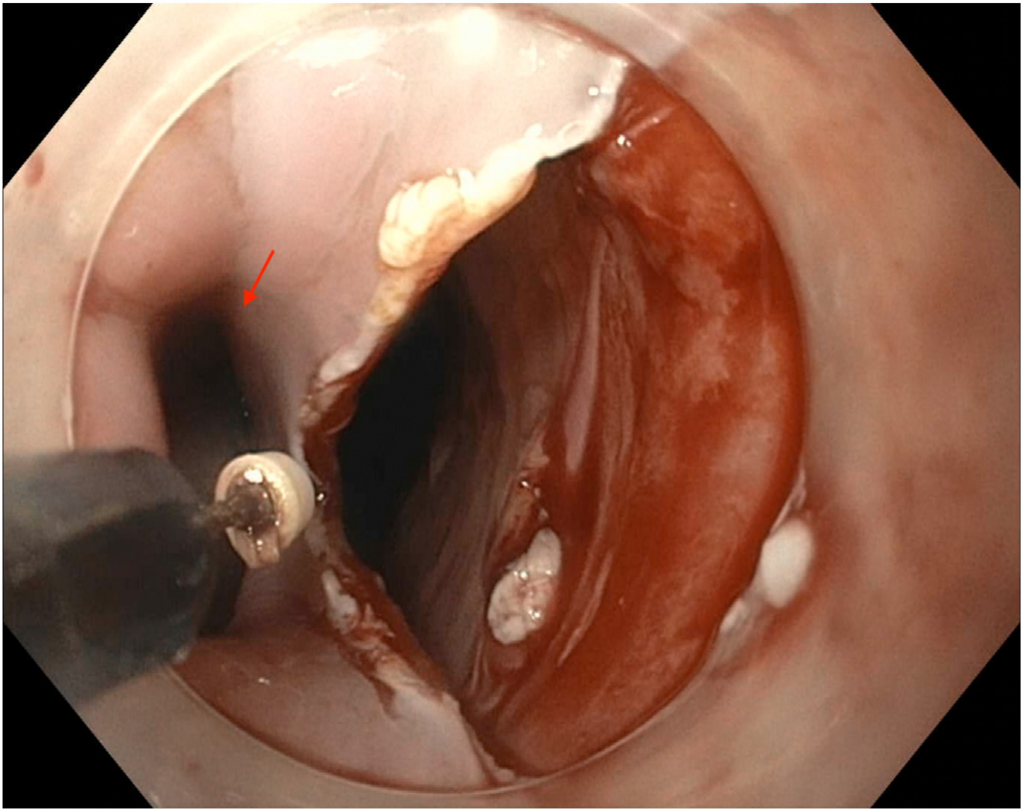
Distal aspect of mucosal septum and mucosotomy. A residual blind-ended pouch from the false lumen can be visualized (indicated by the *red arrow*).

**Figure 7:**
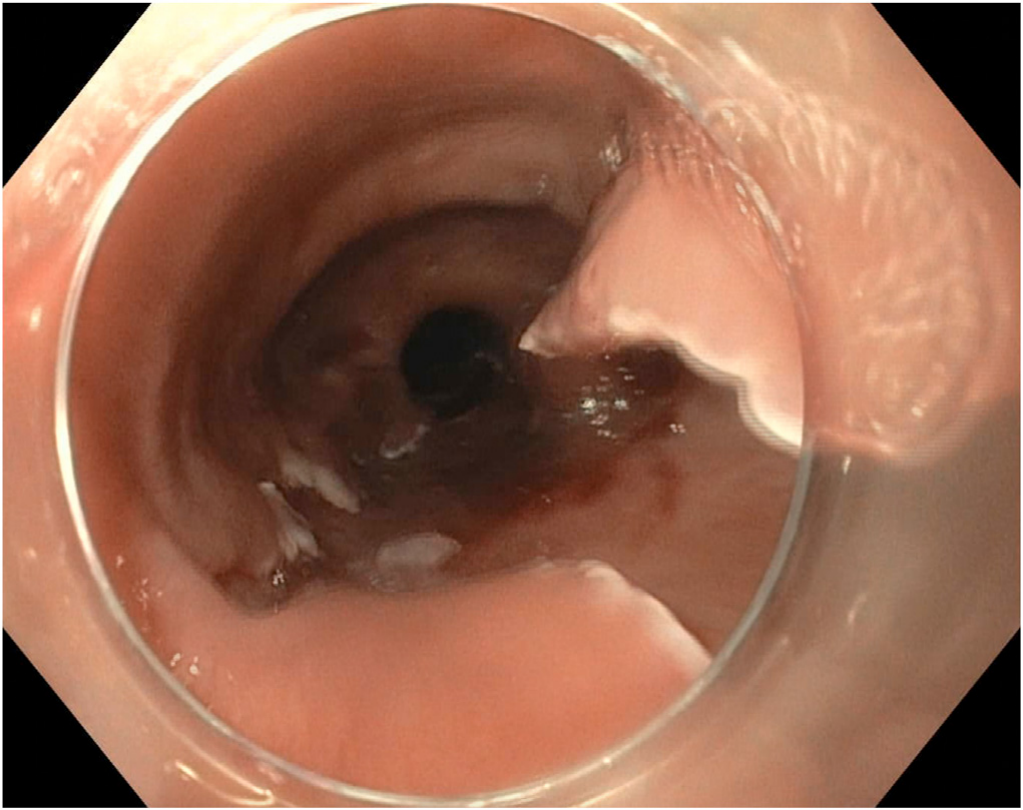
Postmucosotomy appearance of the esophagus, demonstrating a single true lumen and residual portions of the previous mucosal septum.

EGD was completed on postoperative day 44 with the patient under conscious sedation with fentanyl and midazolam. The thoracic surgery team attended the procedure to allow for intraprocedural discussion. A 1- to 2-mm thick mucosal septum spanning the diameter of the esophagus with multiple small (0.5-1 cm) fenestrations between true and false esophageal lumens was identified, extending from 18 to 36 cm from the incisors (Figs. 3 and 4). This appearance was in keeping with an IED. The false lumen accounted for approximately 25% of the circumference of the esophagus along its length. The false lumen was assessed and demonstrated a well-epithelialized, clean cavity with the muscularis propria layer visible and mucosotomy was indicated as the result of this epithelization. This cavity was deroofed using an Olympus IT-nano electrosurgical knife (Olympus America, Center Valley, Pa, USA), using a combination of EndoCut (Erbe VIO 2-2-2) and PreciseSect (6.0) settings (Marietta, Ga, USA). Mucosotomy along the length of the septum, as well as transection of several smaller bridges of mucosal tissue were completed (Figure 5, Figure 6, Figure 7) and a small, distal residual false lumen remained due to proximity to the gastroesophageal junction. Small areas of oozing on the transected flap were treated with hemostatic forceps with complete hemostasis achieved. The duration of the procedure was 20 minutes (Video 1, available online at www.videogie.org).

## Outcome

**Figure 8:**
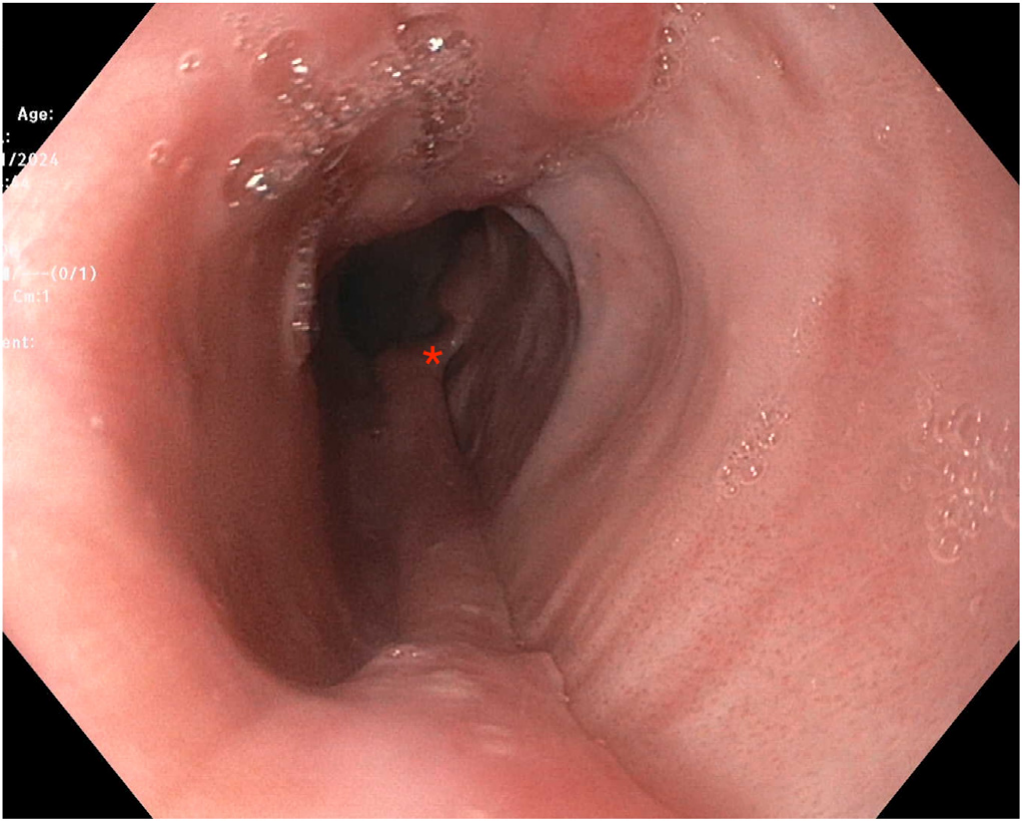
Repeat endoscopy demonstrates healing postmucosotomy and a residual 8-mm pouch at the distal end of the initial mucosotomy (indicated by the *red asterisk*).

**Figure 9:**
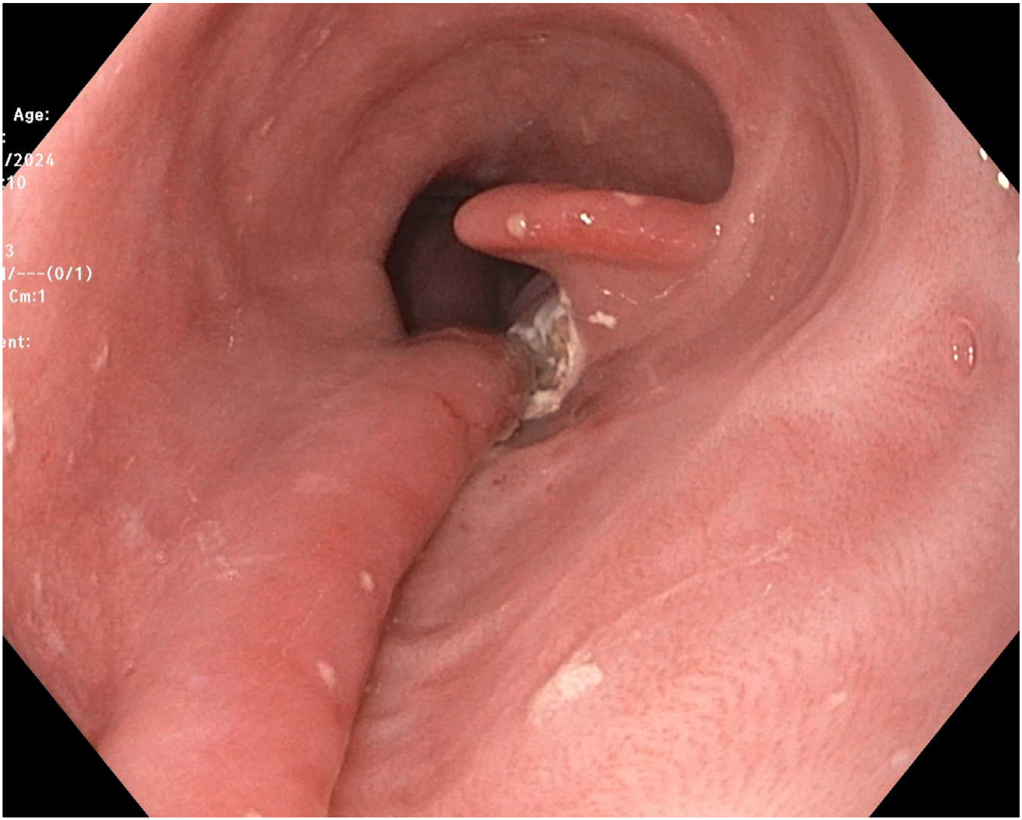
Appearance of the residual pouch after further mucostomy with hot forceps was completed.

The patient reported immediate improvement in esophageal dysphagia postprocedure and tolerated a regular diet within 24 hours. Repeat fluoroscopic esophagram 4 days after the procedure demonstrated a minimal residual false lumen in the distal esophagus not significantly impacting esophageal transit. She took pantoprazole, 40 mg oral daily, for 8 weeks postmucosotomy. She began to notice a esophageal sticking sensation when eating rice and underwent repeat endoscopy 3 months after the initial procedure, which revealed a residual 8-mm pouch at the distal portion of the original mucosotomy (Fig. 8). Further mucosotomy to correct this pouch was completed with hot biopsy forceps, which were chosen to lower cost, without adverse events reported (Fig. 9). The remainder of the esophagus was well-healed and widely patent on this repeat endoscopy, and the patient was asymptomatic immediately after this repeat mucosotomy.

## Patient consent

Patient consent for creation and publication of this case report was obtained both verbally and in a signed document.

## Disclosures

Dr Trasolini is a consultant for Boston Scientific, Medtronic, Fractyl Health, and Fujifilm. The remaining authors had no relevant disclosures to make.

## Supplementary data

Video 1 Intramural esophageal dissection identified on fluoroscopic esophagram and treated with endoscopic electrosurgical mucosotomy.
